# Is it Time to Test and Treat for Syphilis?

**DOI:** 10.1007/s11606-025-09547-x

**Published:** 2025-05-02

**Authors:** Noah Kojima, Jeffrey D. Klausner

**Affiliations:** 1 Department of Population and Public Health Sciences, Keck School of Medicine, University of Southern California, Los Angeles, CA USA; 2 Department of Population and Public Health Sciences, Keck School of Medicine, Los Angeles, CA USA

**Keywords:** sexually transmitted infections, sexually transmitted diseases, syphilis, test and treat

Syphilis is a sexually transmitted infection (STI) caused by *Treponema pallidum* subspecies *pallidum*.^1^ Syphilis has a wide range of clinical symptoms depending on its stage. Severe complications of syphilis include neurosyphilis, cardiovascular syphilis, and congenital syphilis. Additionally, syphilis increases the likelihood of HIV acquisition and transmission, a particular concern among certain at-risk groups.

Syphilis continues to be a problem. Despite calls from the World Health Organization to eliminate mother-to-child transmission of syphilis globally, the number of syphilis cases increased over the last decade. In the USA, the number of reported cases of syphilis increased from 115,000 cases in 2018 to more than 209,000 cases in 2023 including 3882 cases of congenital syphilis, a 50-year high.^1^ We need new approaches to address the ongoing crisis.

The U.S. Department of Health and Human Services developed the STI National Strategic Plan to reverse the recent rise in STIs in the USA.^2^ The goals of the plan are to prevent new infections; improve the health of people by reducing adverse STI outcomes; accelerate progress in STI research, technology, and innovation; reduce STI-related health disparities and health inequities; and achieve integrated, coordinated efforts that address the STI epidemic, with specific priority populations which include adolescents and young adults, men who have sex with men, and pregnant women.

Due to the COVID-19 pandemic and use of home testing, there is increased public awareness of rapid, point-of-care, lateral flow assays. In general, lateral flow assays are disposable diagnostic devices that test for biomarkers in samples, which include saliva, blood, and urine specimens. The benefits of lateral flow assays include simplicity of use, accessible price, and shelf-life stability.

Recent advances in the availability of accurate, 10–15-minute point-of-care syphilis tests have created an opportunity for reconsidering our approach to syphilis (Table 1).^3^ Traditionally, many clinicians have been trained that the diagnosis of syphilis requires both the use of a laboratory-based treponemal and nontreponemal antibody test for the confirmation and staging of infection. Such an algorithm is often associated with a delay in or lack of treatment due to loss-to-follow-up. Treatment at the time of a positive test result, a “test and treat” strategy, could be highly effective and decrease transmission. In January 2024, the National Syphilis Task Force issued considerations for the use of rapid point-of-care syphilis tests including the suggestion for immediate testing and treatment, but clinical uptake continues to be limited.^3^ Patients that could benefit most from this strategy include those with challenges accessing medical care and those at higher risk for loss to follow-up for treatment such as pregnant women, youth, sex workers, and people experiencing homelessness.

**Table 1:** Performance Characteristics of U.S. FDA-Approved Rapid Point-of-Care Syphilis Tests

	Estimate (95% CI)	
Test	Sensitivity	Specificity
Syphilis Health Check*	98.5 (92.1–100.0)	95.9**** (81.5–100.0)
DPP Syphilis Screen & Confirm**	85.9 (81.4–89.6)	100.0 (95.4–100.0)
First To Know***	93.4 (87.0–96.8)	99.5 (98.9–99.8)

^*^Bristow CC, Klausner JD, Tran A. Clinical Test Performance of a Rapid Point-of-Care Syphilis Treponemal Antibody Test: A Systematic Review and Meta-analysis. *Clinical Infectious Diseases*. 2020;71(Supplement_1):S52-S57. 10.1093/cid/ciaa350

^**^(Treponemal component with operator reading) Vargas SK, Qquellon J, Vasquez F, Konda KA, Calvo G, Reyes-Diaz M, et al. Laboratory Evaluation of the DPP Syphilis Screen & Confirm Assay. *Microbiology Spectrum*. 2022 Vol. 10 Issue 3. 10.1128/spectrum.02642-21

***NOWDiagnostics. Package Insert for First To Know. 2024

****Package insert = 97.3%

While there is some concern that immediate treatment based on a rapid point-of-care test alone may result in overtreatment due to prior resolved infection, that concern may be overstated. Persons with a known prior syphilis or treatment history should not be offered rapid point-of-care testing with current tests that only detect treponemal antibodies. Once positive, treponemal antibodies may stay positive for life. In groups at increased risk for syphilis due to sexual practices, the prevalence of treponemal antibodies, however, is only about 25%.^4^

To address the need for quantitative titers to monitor treatment response, treatment may still be initiated among those with a positive rapid test with additional laboratory-based testing over time. Given the high sensitivity and low cost of rapid point-of-care tests, a negative rapid test would obviate the need for further testing and be highly cost-effective.

Treatment for syphilis continues to be highly successful. Patients with primary, secondary, or latent syphilis of less than 12 months duration should be treated with one intramuscular injection of 2.4 million units of Penicillin G benzathine. Those with late latent (or an unknown duration latent syphilis) should be given the same dose weekly for 3 weeks. The rationale for the longer duration of treatment in late latent infection is based on expert opinion,^5^ stated nearly 50 years ago. Three doses might not be necessary, but evidence is needed to update that guidance.^6^ Importantly, a single dose of Penicillin G benzathine injection prevents both mother-to-child and sexual transmission^1^. For people that cannot tolerate Penicillin G benzathine or when Penicillin G benzathine is unavailable, doxycycline or ceftriaxone are appropriate alternative medications.

Given the severity of the current syphilis crisis and the public health benefits of immediate treatment to reduce further transmission, it is time to advance clinical practice to a “test and treat” model for syphilis. This strategy would lead to increased numbers of people tested for syphilis, treated for syphilis, and decrease either mother-to-child or person-to-person transmission. Furthermore, research is urgently needed to determine if the standard practice of repeated doses to treat latent infection is beneficial.
